# Anesthetic Management of a Patient With Hereditary Spastic Paraplegia: A Case Report

**DOI:** 10.1155/cria/8487425

**Published:** 2026-05-27

**Authors:** Isbi Sotelo, Kevin R. Olsen

**Affiliations:** ^1^ Department of Anesthesiology, BayCare Medical Group, 300 Pinellas Street, Tampa, 33756, Florida, USA

**Keywords:** anesthesiology, hereditary spastic paraplegia, neurodegenerative disease, neuromuscular disease

## Abstract

**Background:**

Hereditary spastic paraplegia is an extremely rare, heterogenous, inherited, progressive neurologic disorder characterized by lower‐extremity weakness and spasticity in pure forms, with complex forms including symptoms such as seizures, dementia, and ataxia. Due to its rarity, evidence guiding anesthetic management is limited, consisting of only eight published case reports.

**Case Report:**

We report the anesthetic management of a patient with pure hereditary spastic paraplegia undergoing renal mass cryoablation in the prone position. General endotracheal anesthesia was induced with propofol, maintained with isoflurane, and included nondepolarizing neuromuscular blockade with reversal using sugammadex.

**Conclusion:**

Patients with hereditary spastic paraplegia typically have experienced multiple anesthetic exposures prior to diagnosis, suggesting that standard anesthetic techniques are generally well‐tolerated in this population without significant adverse effects. Given that pure HSP is an upper‐motor neuron disorder—without involvement of the neuromuscular junction—extensive disease‐specific modifications to anesthetic plans are likely unnecessary in the absence of additional comorbid risk factors.

## 1. Introduction

Hereditary (familial) spastic paraplegia (HSP/FSP), also known as Strumpell–Lorrain disease, is a rare, inherited, heterogenous, group of progressive neurologic disorders with more than 80 genetic types. It is characterized in its pure form, as the name suggests, by progressive weakness and spasticity of the bilateral lower extremities, hypertonic urinary bladder disturbances, and pallhypesthesia [1]. In its complex or complicated form, other neurologic symptoms may appear such as seizures, ataxia, muscle atrophy, intellectual disability/dementia, and peripheral neuropathy [1]. Pathologically, HSP results in defects that predominately involve the degeneration of corticospinal and dorsal spinal tracts resulting in axonal atrophy. Importantly, the pure form of HSP is classified as an upper‐motor neuron disorder; the lower motor neurons, neuromuscular junctions, and skeletal muscle fibers themselves remain functionally intact, whereas in the complex forms, lower motor neuron symptoms may also appear. The worldwide incidence is estimated to be 0.9 to 9.6 per 100,000 persons—making this a rather rare entity [2]. Knowledge among anesthesiologists about the existence of this disease as well as evidence informing best practices for anesthetic management is severely limited, with only 8 case reports (5 utilizing general and 3 utilizing neuraxial anesthesia) in the English literature to date [3–9]. This understandable ignorance among anesthesiologists may lead to unnecessary withholding of certain medications or techniques out of an “abundance of caution” that may lead to nonoptimal care or even harm for the patient—such as avoidance of normal doses of nondepolarizing neuromuscular blockers, hesitancy to use neuraxial techniques, and restricting malignant hyperthermia triggering agents—all for the fear of potential complications that are seen with other neuromuscular diseases. Here, we hope to expand the literature to show how HSP can be managed safely in the perioperative space. Written Health Insurance Portability and Accountability Act authorization has been obtained from the patient.

## 2. Case Description

A 71‐year‐old 87‐kg male with a history of autosomal recessive “pure” HSP first diagnosed at age 61 with primary symptoms of urinary urgency, progressive lower extremity spasticity/weakness and distal bilateral upper and lower extremity pallhypesthesia presented with a newly discovered renal mass concerning for renal cell carcinoma and planned to undergo mass cryoablation with interventional radiology. He had previously been misdiagnosed with cervical spinal stenosis in his early 50s and underwent a general anesthetic for anterior cervical discectomy and fusion without issue anesthetically but also without relief of his progressive symptoms. He was subsequently misdiagnosed with both amyotrophic lateral sclerosis followed by myasthenia gravis in his late 50s and reportedly underwent colonoscopy and endoscopy with propofol without issues. Recently, he was diagnosed with renal cell carcinoma and scheduled to undergo a CT‐guided cryoablation procedure with interventional radiology in the prone position—necessitating a general anesthetic. Given his history of uneventful anesthetics prior to obtaining a diagnosis of HSP, we postulated that he should have no issues with a “routine” general anesthetic for his upcoming procedure, consisting of inhaled volatile anesthetic and nondepolarizing neuromuscular blockade.

Routine preoperative lab work consisting of a complete blood count and basic metabolic panel showed no significant abnormalities. A 20G intravenous (IV) catheter was placed in the preoperative area and attached to a liter bag of Lactated Ringers. Upon arrival to the CT suite, ASA standard monitors were placed and IV antiemetics were administered, including 20 mg of famotidine and 4 mg of ondansetron. The patient was preoxygenated with 1.0 FiO2 with the anesthesia circuit for 5 min, and general anesthesia was induced via Peripheral IV with 100 mg of lidocaine, 100 mcg of fentanyl, 130 mg of propofol, and 50 mg of rocuronium. Endotracheal intubation utilizing video laryngoscopy was easy, and placement of a standard 7.5 mm cuffed endotracheal tube was uneventful. General anesthesia was maintained using isoflurane with an end‐tidal concentration between 0.5% and 0.9% in oxygen and air with an FiO_2_ of 0.6. Neuromuscular blockade was assessed every 10 min using a qualitative peripheral nerve stimulator on the orbicularis oculi. This location and technique were selected due to the patient’s position and quantitative train‐of‐four (TOF) being unavailable at this operating suite. The patient was then placed in the prone position with a prone pillow and bilateral arms tucked. The eyes and face were checked every 15 min for pressure and appropriate positioning. Ephedrine was occasionally bolused to keep mean arterial pressure > 70 mmHg, for a total of 15 mg ephedrine given throughout the duration of the case. Due to the quick nature of the procedure, no additional rocuronium was given. The total procedure time was approximately 91 min from induction to extubation. The initial TOF count was 0/4 at 10 min postinduction, with gradual recovery to 1/4 at approximately 30 min and spontaneous recovery to 4/4 occurring at approximately 65 min postadministration of rocuronium. The duration of neuromuscular blockade did not appear prolonged relative to what would be expected with a 50‐mg dose of rocuronium in a patient without neuromuscular disease. By the end of the procedure, the patient had spontaneous recovery of his qualitative TOF to 4/4. Given the patient’s neuromuscular disease and lack of quantitative TOF monitoring, the decision was made to antagonize any remaining rocuronium with 200 mg of sugammadex (approximately 2.3 mg/kg dose)—consistent with the ASA practice guideline for monitoring and antagonism of neuromuscular blockade (2 mg/kg dose) [10]. Within 5 min of sugammadex administration, TOF again showed 4/4 with sustained tetany for 5 s on the orbicularis oculi and confirmed with a TOF 4/4 and sustained tetanus on the adductor pollicis muscle. The patient was extubated uneventfully after following commands including bilateral squeezing of hand, > 5 s head lift, demonstrated tidal volumes > 8 mL/kg, responded to their name and had a strong cough. The patient was discharged the same day. A total of 600 mLs of Lactated Ringers were administered with an EBL of 10 mL.

## 3. Discussion

The optimal safe anesthetic management of patients with HSP, in its many forms, is unknown with sparse literature and only 8 cases reports in the English literature. Due to its rarity and lack of evidence‐based recommendations on management, anesthesiologists often take precautions that they would otherwise employ for other neuromuscular diseases. These precautions may include avoidance of typical doses of nondepolarizing neuromuscular blocking drugs to potentially avoid prolonged paralysis, avoidance of depolarizing neuromuscular block drugs for fear of triggering malignant hyperthermia or hyperkalemia, avoidance of neuraxial or regional techniques to avoid possible worsening of symptoms and possibly even avoidance of volatile anesthetics.

Current evidence gleaned from an understanding of the pathophysiology, and these case reports have not demonstrated exaggerated sensitivity to typical doses of non‐depolarizing agents and detail appropriate reversal of these agents with both sugammadex and the combination of glycopyrrolate and neostigmine. Likewise, neuraxial and regional anesthetic techniques have been used without documented deterioration of neurologic status or perioperative complications. Furthermore, a variety of inhaled and IV agents have been reported on, all without any indication of complications attributable to the anesthetic of choice. Although these experiences are limited, and often fail to distinguish whether the patients are suffering from pure or complex HSP, they collectively suggest that many of the commonly utilized or proposed precautions may be unnecessary. Furthermore, it is probable that many HSP patients undergo a variety of anesthetics prior to receiving their diagnosis, given the frequently delayed and circuitous diagnostic course, as illustrated by our patient’s multiple misdiagnoses over a decade and are thus exposed uneventfully to many different agents. As with any neuromuscular disorder, individualized assessment is essential, but routine exclusion of depolarizing neuromuscular blockers, volatile anesthetics, or neuraxial techniques does not appear to be supported by existing evidence or by the known pathophysiology of HSP.

Continued reporting of anesthetic experiences in HSP is needed to refine practice and reduce unnecessary caution. Until larger datasets are available, anesthesiologists should consider the mechanistic distinctions between HSP and other neuromuscular diseases and avoid overgeneralizing restrictions that may complicate care without improving safety.

## Author Contributions

Kevin R. Olsen, MD: This author participated in patient care and helped write the manuscript. Isbi Sotelo, CAA: This author participated in patient care and helped write the manuscript.

## Funding

No funding was received for this manuscript.

## Conflicts of Interest

The authors declare no conflicts of interest.

## Data Availability

The data that support the findings of this study are available from the corresponding author upon reasonable request.
